# Public Health Emergency Preparedness and Response Communications with Health Care Providers: A Literature Review

**DOI:** 10.1186/1471-2458-11-337

**Published:** 2011-05-18

**Authors:** Debra Revere, Kailey Nelson, Hanne Thiede, Jeffrey Duchin, Andy Stergachis, Janet Baseman

**Affiliations:** 1 Department of Health Services, University of Washington, Seattle, WA, USA; 2 Department of Epidemiology, University of Washington, Seattle, WA, USA; 3 Communicable Disease Control, Epidemiology & Immunization Section, Public Health-Seattle & King County, Seattle, WA, USA; 4 Department of Global Health, University of Washington, Seattle, WA, USA

## Abstract

**Background:**

Health care providers (HCPs) play an important role in public health emergency preparedness and response (PHEPR) so need to be aware of public health threats and emergencies. To inform HCPs, public health issues PHEPR messages that provide guidelines and updates, and facilitate surveillance so HCPs will recognize and control communicable diseases, prevent excess deaths and mitigate suffering. Public health agencies need to know that the PHEPR messages sent to HCPs reach their target audience and are effective and informative. Public health agencies need to know that the PHEPR messages sent to HCPs reach their target audience and are effective and informative. We conducted a literature review to investigate the systems and tools used by public health to generate PHEPR communications to HCPs, and to identify specific characteristics of message delivery mechanisms and formats that may be associated with effective PHEPR communications.

**Methods:**

A systematic review of peer- and non-peer-reviewed literature focused on the following questions: 1) What public health systems exist for communicating PHEPR messages from public health agencies to HCPs? 2) Have these systems been evaluated and, if yes, what criteria were used to evaluate these systems? 3) What have these evaluations discovered about characterizations of the most effective ways for public health agencies to communicate PHEPR messages to HCPs?

**Results:**

We identified 25 systems or tools for communicating PHEPR messages from public health agencies to HCPs. Few articles assessed PHEPR communication systems or messaging methods or outcomes. Only one study compared the effectiveness of the delivery format, device or message itself. We also discovered that the potential is high for HCPs to experience "message overload" given redundancy of PHEPR messaging in multiple formats and/or through different delivery systems.

**Conclusions:**

We found that detailed descriptions of PHEPR messaging from public health to HCPs are scarce in the literature and, even when available are rarely evaluated in any systematic fashion. To meet present-day and future information needs for emergency preparedness, more attention needs to be given to evaluating the effectiveness of these systems in a scientifically rigorous manner.

## Background

**Figure 1:**
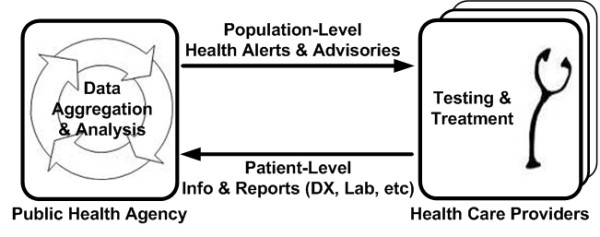
**HCP-Public Health message and information exchange**.

Public health emergency preparedness and response (PHEPR) involves activities directed at preventing possible emergencies and planning to ensure an adequate response and recovery if an emergency occurs. The public health system itself is a complex network of organizations and individuals that work together for the benefit of the public's health. These entities include public health agencies at local, state and federal levels, public safety agencies, emergency managers, academia, business, communities, the media, and the healthcare delivery system [1]. As one component of the PHEPR system, information contributed by health care providers (HCPs) to public health is aggregated, analyzed and used by public health agencies, in part, to inform early event detection and situational awareness [2]. Figure 1 illustrates a simplified transfer of information from HCPs to public health which is aggregated, analyzed and used to inform public health alerts and advisories which are sent to HCPs.

The importance of the transmission of HCP information to public health, particularly for notifiable condition reporting, has been well-documented [2-5]. HCPs serve a critical role in public health's recognition and control of communicable diseases as illustrated by West Nile Virus [6] and SARS [7]; influenza and influenza-like illness [8]; foodborne illnesses [9]; and illnesses associated with intentional release of biologic agents such as anthrax [10,11]. In public health responses involving bioterrorism, HCPs have an especially important role since they will likely report such cases of unexplained or unusual illness to state and local public health officials who, in turn, may be able to conduct investigations and identify specific epidemiologic patterns or characteristics potentially indicative of bioterrorism [12].

During an emergency situation health care providers (HCPs) are depended on to prevent excess deaths, treat the injured, and mitigate suffering [13]. To do this, and given that individuals will seek medical care in multiple locations during an emergency, HCPs need to be aware of public health threats and emergencies, issue guidelines and updates, and facilitate surveillance [14]. On September 11, 2001, when telephone and paging systems failed, the New York City Department of Health and Mental Hygiene successfully used email and fax to distribute public health broadcast alerts to all NYC emergency departments, commercial and hospital laboratories, infection-control programs, and select providers [15]. In an emergency, effective communication will not only depend on the information/message, but on the type of communication system or tool, the delivery format, and the robustness of the system.

While timely, efficient, and effective communications between public health and HCPs is an important part of public health emergency preparedness and response (PHEPR), most publications concerned with this exchange have emphasized the HCP-to-public health component. Yet, it is well-established that the "return" of information to HCPs is also significant. We conducted a systematic literature review to investigate the systems and tools used by public health to generate PHEPR communications to HCPs, and to identify specific characteristics of message delivery mechanisms and formats that may be associated with effective PHEPR communications.

## Methods

Three questions guided this literature review:

What public health systems exist for communicating PHEPR messages from public health agencies to HCPs?

Have these systems been evaluated and, if yes, what criteria were used to evaluate these systems?

What have these evaluations discovered about characterizations of the most effective ways for public health agencies to communicate PHEPR messages to HCPs?

Table 1 lists the subject terms and keyword terms identified for key concepts for the search. To ensure retrieval of different types of PHEPR messages we included both health alerts (messages of the highest level of importance that warrant immediate action or attention) and health advisories (messages that provides key information for a specific incident or situation, such as a guideline change, and might not require immediate action). We also included as search terms any system, communication method or device that facilitated these communications.

**Table 1:** Search term categories

Occupation/Discipline	Event	Activity
"healthcare providers" OR "health care providers"	disaster	communication

doctors OR physicians	"disease outbreak"	"disease event"

nurses	emergency	"emergency alert"

pharmacists	response	"emergency communication"

"public health"	pandemic	"health alert" OR "public health alert"

veterinarians	preparedness	"health advisory"

	terrorism	"preparedness message"

	surveillance	"preparedness communication"

Public health literature is reported to be poorly indexed in bibliographic databases and dispersed across a wide variety of journals and other sources, as well as across many disciplines [16]. We included "grey" or non-peer-reviewed literature sources [17] to ensure wide coverage of less accessible materials such as government reports and conference proceedings (Table 2).

**Table 2:** Resources searched

Bibliographic, Peer-Reviewed Literature Sources	
CINAHL^®^: nursing & allied health literature	

National Library of Medicine's MEDLINE^® ^through the PubMed^® ^interface	

INSPEC^®^: computer & information technologies	

Web of Science^®^: science & social science journals & cited references	

**Non-Peer-Reviewed Literature Sources**	

Agency for Healthcare Research & Quality website	CDC website

GPO Access	National Academies Press

NLM Gateway: biomedical books & meeting abstracts	RAND website: policy & decision-making

RWJ publications	Google search engine

*scirus*: scientific specialty search engine for pre-prints, gov't & institutional repositories	

The exact search terminology used was tailored for each database as appropriate to its structure and thesaurus to ensure a high degree of sensitivity (Table 3).

**Table 3:** Search Strategies

Peer-Reviewed Literature	
CINAHL	("public health") AND (doctors OR physicians OR nurses OR pharmacists OR veterinarians OR "healthcare providers" OR "health care providers" OR surveillance) AND (communication OR "emergency communication" OR "disease event" OR "health alert" OR "public health alert" OR "emergency alert") AND (emergency OR disaster OR terrorism OR pandemic OR preparedness OR response OR "disease outbreak")
MEDLINE	

INSPEC	("public health" OR "emergency services" OR "emergency preparedness" OR "emergency planning" OR "surveillance activity" OR "emergency response") AND alert

Web of Science	("public health" AND (doctors OR physicians OR nurses OR pharmacists OR veterinarians OR "healthcare providers" OR "health care providers" OR surveillance) AND (communication OR "response capacity" OR "emergency communication" OR "disease event" OR "health alert" OR "public health alert" OR "emergency alert") AND (emergency OR disaster OR terrorism OR pandemic OR preparedness OR response))

Snowball technique	hand-searching article references, related records, tables of contents of pertinent journals

**Grey Literature**	

AHRQ	"public health" AND "emergency preparedness" AND alert
CDC	"public health" AND "emergency preparedness" AND "emergency communication"
GPO Access	"public health" AND providers* AND communication AND emergency
National Academies Press	"public health" AND terrorism AND alert
NLM Gateway	"public health" AND "bidirectional communication" AND "health alert"
RAND	"public health" AND disaster AND providers* AND alert
RWJ publications	
*www.google.com*	
*www.scirus.com*	[*providers also listed individually by provider type, e.g., nurses, etc.]

The Web of Science^® ^database was used to conduct cited reference searches of relevant articles. In addition, we hand-searched (known as snowball sampling) the reference lists of relevant articles and the tables of contents of the following journals: *Journal of Homeland Security and Emergency Management, Disaster Medicine & Public Health Preparednes*s, and *American Journal of Disaster Medicin*e.

**Figure 2:**
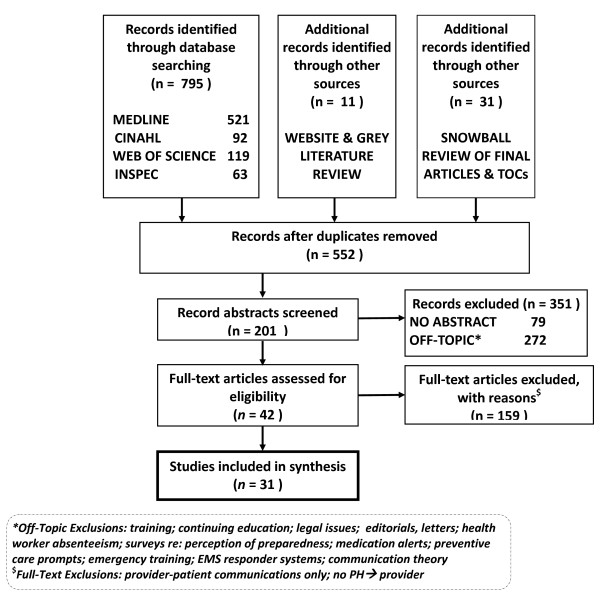
**Flowchart of literature review process**.

The review was limited to publications in the English language and to materials published between 01/2000 through 01/2011. All search strategies were recorded at each step. Citations from database searches were downloaded into the EndNote bibliographic reference program (http://www.endnote.com/) or manually entered as needed. Duplicates were removed. Figure 2 illustrates the identification, screening, eligibility and inclusion numbers, and rationale for excluded materials in our search and selection process [18].

Articles were included if they described systems or tools for public health agencies to communicate PHEPR messages to HCPs or included an evaluation of these systems or tools. Data extracted from the articles included: purpose, location, organization or agency involved, HCP population, method(s) of communication, and type of evaluation performed, if conducted. If an evaluation was performed, the outcomes were extracted.

## Results

Of the initial set of 42 full-text articles assessed for eligibility, 11 were excluded once read as they only described systems that sent PHEPR messages to health departments (*n *= 6) or were opinion articles (*n *= 5). Data extraction from the final 31 articles resulted in identification of 25 different systems, with one article describing more than one system. Overall, the final 31 articles contained information on the purpose of the system or tool (100%), location of the system (100%), public health organization or agency involved (100%), targeted HCP population (100%), and method(s) used by public health to communicate PHEPR messages to HCPs (100%).

Eleven articles (covering 9 systems) included a description of the evaluation used with the system. Type of evaluations included comparative [19], interviewing [20], surveying [21,22], retrospective [23,24], formative [25], and an assessment following a simulation exercise [26]. One article reported a causal relationship could be "inferred" between the dissemination of health advisories and HCP reporting and testing [27] and two reported receiving feedback but did not detail method [28,29]. The remaining articles (65%) either did not mention an evaluation or did not contain enough information to determine if an evaluation had been conducted.

Of the 25 systems and tools documented, the majority (96%) were North America-based. The location of the systems included: 40% state-level, 32% city-level, 16% country-level, and 8% regional, with one international system (4%). Only one tool was designed to provide PHEPR messages to veterinarians; the remaining targeted HCPs in hospitals, emergency departments and/or outpatient clinical settings.

The majority of systems used email (64%) to deliver PHEPR messages. Systems also delivered messages by phone, including cellular (36%); fax (36%); pager (28%); SMS text messaging (16%); handheld devices such as PDAs or Blackberry^® ^(16%); other devices such as radios (16%); messaging through an electronic medical record system (12%); and "social media" (4%). Some systems also posted the PHEPR message to a web site (24%) for passive consumption. A majority of systems used more than one method (60%) for delivering messages. Only 4 systems were described in sufficient detail to determine that each method was attempted sequentially as opposed to redundant messages being delivered through all devices and formats. Table 4 (additional file 1 table S1) lists each messaging system or tool included in the final retrieval set and indicates type of evaluation conducted where applicable.

## Discussion

After conducting a systematic search, we identified 25 systems or tools currently being used to communicate PHEPR messages from public health to HCPs. Of the 9 systems that reported an evaluation, only 2 provided sufficient detail of methodology used. During a Q fever outbreak, two public health alert faxes were sent asking physicians to submit serum samples on any patient meeting a clinical case definition of Q fever and an association with the area where the outbreak occurred. By examining laboratory reports, Van Woerden et al (2006) found a statistically significant difference between the number of patients tested for Q fever in the target population after the alerts had been sent as compared to a comparable two-week period one year before [19]. Another study retrospectively examined recommended public health agency actions communicated to HCPs through a pop-up in an electronic health record in comparison with lab orders and treatment guidelines and found that a causal relationship "could be inferred" (although with no detail to document this inference) between the alert and a change in HCP behavior [27].

Other system evaluations lacked adequate detail to determine the extent of evaluation activities. Prior to developing GermWatch, a system focused on communicating advisories regarding respiratory viral pathogens and pertussis, Gesteland et al (2007) conducted a formative evaluation of the feasibility and sustainability of the system [25]. However, formative studies, though useful in the planning and early development phases of a system, need to be followed up with an evaluation focused on identifying changes in outcome or performance measures, results, or effectiveness criteria that can be confidently attributed to the system rather than other factors and conditions. While reports of retrospective evaluations of ProMED, a global outbreak surveillance system [23,24], the messaging tools used in conjunction with a TOPOFF exercise [26], and a survey of homeless service providers during the SARS outbreak in Toronto [20] identify problems and propose measures to counteract problematic communications issues between public health and HCPs, the reports lacked the detailed methodology or results that are needed to assess the rigor of these evaluations.

One of the most widespread strategies in the U.S. for public health agencies to communicate to HCPs on both national and local levels is through the CDC's Health Alert Network (HAN) program which communicates information about infectious disease outbreaks and public health implications of national disasters within its health alerts, advisories, and updates [14,29,30]. Given its wide coverage, we were surprised to find so few studies attempting to systematically verify that HAN messages are received, processed, and/or acted upon by the intended recipients outside of public health agencies. As a result, in part, of current studies of the 2009 H1N1 outbreak, we are now learning that PHEPR messages may not be reaching their targeted audiences. For example, results of a cross-sectional survey of health departments, physicians, and pharmacists in Kentucky regarding information dissemination and receipt during the early H1N1 outbreak found that deficiencies exist in the effectiveness of public health PHEPR communications to HCPs. While 81% of responding local health departments (LHDs) rated their capacity to disseminate information to HCPs as very good or excellent, only 52% of surveyed physicians and 16% of surveyed pharmacists reported receiving any information about H1N1 from a LHD. Seventy-four percent of pharmacists were not aware of their LHD's emergency plan in the event of an influenza outbreak [31].

In conducting this review we discovered that there are multiple sources from which HCPs may receive HAN communications. CDC not only sends messages to state and local public health agencies that then disseminate to HCPs, but clinicians can also sign up to receive HAN messages directly through the CDC's Clinician Outreach Communication Activity (COCA) as well as through any of the 176 COCA partner organizations that pass on or post COCA-generated notices of new and updated CDC information on emerging health threats [21,22]. While any PHEPR situation presents challenges in communicating about uncertainties, collaborating across and within organizations, and communicating timely messages [32], every additional messaging source raises the potential for redundant and conflicting information. COCA disseminates updates bi-weekly (more frequently when there is emergency information or event-specific updates). Excluding HAN alerts, a tally of messages disseminated through COCA from 2008-2010 yielded 140 messages that each contain as many as 7 topical messages.

Avoiding the communication of multiple and redundant messages that can engender "alert overload" in HCPs is important, especially in a public health emergency situation. The HAN system allows HCPs to set a preference for receiving messages but, as mentioned above, if the HCP is receiving messages from different sources the redundancy potential increases. Staes et al (2011) presented an objective analysis of communication between public health agencies, health care organizations, and frontline HCPs during the 2009 H1N1 outbreak. The investigators conducted a cross-sectional survey to understand communication processes between public health and frontline HCPs and found that HCPs received redundant messages; were challenged to keep up with evolving and tailored messages from multiple organizations at a time when clinic volumes, patient concerns, and media exposure were increasing; and were overwhelmed by e-mail volume. The study suggests that PHEPR messages sent to HCPs be concise and clearly identified [33].

We found there are numerous formats (email, fax, etc) in which to deliver PHEPR messages to HCPs. When more than one format was available it was not clear if HCPs were given a choice between different ways to receive messages as opposed to receiving redundant messages in different formats or through different delivery systems. Allowing HCPs to set preferences for receiving PHEPR messages might improve response.

Our review has three main limitations: 1) scope and search terms; 2) access to full-text articles; and 3) lack of data in the included articles. For practical reasons we limited ourselves to materials written in the English language. While we did not limit ourselves to U.S. systems or studies, it is possible that systems of PHEPR messaging to HCPs developed in Europe and Asia may be written in other languages. It is also possible that our search strategy did not cast either a wide or targeted enough net to capture relevant literature. Perhaps modifications to the terminology or concept operators would have yielded better retrieval sets. We were limited to resources accessible through our academic libraries and their inter-library partnerships so may have missed some material. Another limitation is our elimination of articles missing or with uninformative abstracts. Again, it is possible that this omitted key articles from our results. Lack of data was an issue as many articles did not contain sufficient descriptive information. Despite these limitations, our results show that detailed descriptions of PHEPR messaging from public health to HCPs are scarce in the literature and, even when available are rarely evaluated in any systematic fashion.

## Conclusions

This review shows that little is known about the effectiveness of PHEPR communications from public health to HCPs. We also found that by using multiple formats and delivery methods, current systems and tools may be increasing, rather than reducing, communication challenges for HCPs with unnecessarily redundant messages; confusion due to messages that may reflect conflicting federal, state and local guidelines, information and concerns; alert "overload"; and lack of tailored preferences for receiving these important messages.

Much has been written about the "astute clinician" who noted an unusual clinical finding and set off the public health alarm concerning the first case of anthrax in Palm Beach County, Florida in October 2001 [34]. Given the importance of HCPs in PHEPR, more research needs to be done to further investigate how public health can communicate effectively with HCPs. There are numerous questions about these systems and tools that need to be answered, some basic, such as: Have PHEPR messages been successfully delivered? Were they read and, if yes, can the date or time of their delivery and their content be recalled? Is there an optimal frequency for sending PHEPR messages? What components of a message are most important for the message to be perceived as credible, authoritative, complete? What impact do PHEPR messages have on HCP behavior, surveillance or reporting of suspected or confirmed events of public health interest or PHEPR knowledge?

One example of new research being conducted in this area is the REACH Trial in which the authors are using a randomized, community-based trial method to investigate the effectiveness of various message delivery systems (email, fax, and SMS) for communicating PHEPR messages from public health agencies to HCPs [35]. The primary aim of REACH is to determine the effectiveness of various message delivery systems (email, fax, and SMS) for communicating PHEPR messages from public health agencies to HCPs and to compare the effectiveness of communication methods between these two groups across diverse communities. This is however, only one effort. To meet present-day and future information needs for emergency preparedness, concentrated attention needs to be given to evaluating the effectiveness of PHEPR systems in a scientifically rigorous manner [36].

## Competing interests

The authors declare that they have no competing interests.

## Authors' contributions

DR conceived of and led the search, evaluation and synthesis components. KN participated in the database searches and retrieval set evaluation. DR authored the overall manuscript with contributions by KN, JB, AS, HT and JD. All authors read and approved the final manuscript.

## Pre-publication history

The pre-publication history for this paper can be accessed here:

http://www.biomedcentral.com/1471-2458/11/337/prepub

## Supplementary Material

Additional file 1 **Table S1: Literature selected**. Click here for file
