# Supplementary material for: Public Health Emergency Preparedness and Response Communications with Health Care Providers: A Literature Review
Source: BMC Public Health. 2011 May 18;11:337. doi: 10.1186/1471-2458-11-337 (PMC3121631; doi:10.1186/1471-2458-11-337)
Supplement: Additional file 1 — Table S1: Literature selected. [file 1471-2458-11-337-S1.DOC]

## Table S1. Literature selected

| **Literature Reporting Evaluation** | | | | | | | | | |
| --- | --- | --- | --- | --- | --- | --- | --- | --- | --- |
| **System or Tool** | | **Organization** | **Location (level)** | **Message Delivery** | | **Target HCP Population** | | **Evaluation** | |
| Local system[19] | | Newport City Council Environmental Health Dept | South Wales, UK  (city) | fax | | General HCP | | Comparative study of HCPs who received fax vs no-fax | |
| Unspecified local system[20] | |  | Toronto  (city) | email | | HCPs and others providing homeless services | | Interview providers re: improvements to communication of infection control and quarantine for homeless populations | |
| Clinician Outreach Communication Activity (COCA)[21-22] | | CDC | USA  (national) | email | | MDs, nurses, PAs, pharmacists, paramedics, veterinarians, epis, PH practitioners, state & local HD officials | | Survey re: feedback on coordination with clinicians during H1N1 outbreak | |
| ProMed[23-24] | | Program for Monitoring Emerging Diseases | Interna-tional | email | | HCPs who wish to be alerted to outbreaks of infectious diseases and acute exposures to toxins that affect human health, including those in animals and in plants grown for food or animal feed | | Retrospective evaluation re: informing SARS outbreak | |
| GermWatch[25] | | Univ of UT, UT DOH, Intermountain Healthcare | Utah  (state) | email, web site | | HCPs affiliated with Intermountain Healthcare | | Formative evaluation re: awareness and attitudes about usefulness, access and intent to use population-based data related to respiratory pathogens | |
| Bioterrorism Preparedness  Web Application[26] | | State of CT Dept of Public Health | Connect-icut  (state) | email, pager, cell phone, web site | | Hospital HCPs | | Assessment following Simulation/TOPOFF Exercise identified needed improvements in communication strategies and use of consistent language to streamline communications | |
| Best Practice Advisory(BPA)[27] | | NYC DOHMH | NYC  (city) | pop-up HAN alert in EMR | | HCPs using the Epic Ambulatory EHR | | Causal relationship "inferred" between BPA and improved reporting and testing considerations | |
| Boston Syndromic Surveillance System (B-SYNSS)[28] | | Boston Public Health Commission | Boston  (city) | web site | | HCPs and acute care providers | | "Feedback" re: posting of alerts helpful to HCPs; relevant staff receive info in a timely manner; increasing frequency of communication reinforced reporting requirements and increased awareness of public health priorities among HCPs | |
| Chicago HAN[29] | | Chicago Dept of Public Health | Chicago  (city) | email, pager phone, SMS, handhelds | | Infectious disease MDs, infectious control practitioners, Emergency Dept, pediatricians, hospital laboratories, hospital risk management, patient safety | | Assessed (no method detailed) HAN use during H1N1 response. Findings: HAN helped CDPH expand reach beyond usual hospital users into the community-based medical community; HAN hospital users "pleased" with timeliness and content of H1N1 alerts; hospital-based HAN members distributed alerts to their communities using other communication methods (methods not detailed) | |
| **Literature included but not reporting Evaluation** | | | | | | | | | |
| **System or Tool** | **Organization** | | | | **Location (level)** | | **Message Delivery** | | **Target HCP Population** |
| CityWatch[30] | Department of Health | | | | Illinois  (state) | | email, fax, pager, phone, PDA, other devices | | MDs, nurses, local HDs, PH directors, epidemiologists |
| DOCS4DOCS[38] | Marion County HD, Regenstrief/Indiana University | | | | Marion County, IN  (regional) | | EMR messaging | | HCPs in Health Information Exchange |
| e-clinician[39] | NYC DOHMC, Community Health Care Association of New York State (CHCANYS), Clinical Directors Network, Inc. (CDN) | | | | NYC  (city) | | customized PDA | | HCPs in NYC Community Health Centers |
| govdelivery.com[40] | OR Dept of Human Services, CO Dept of Public Health & Environment, IN Dept of Public Health, AS Dept of Health & Social Services, NE Dept of Health and Human Services, MN Dept of Public Health | | | | Oregon, Colorado, Indiana, Alaska, Nebraska, Minnesota  (state) | | email, SMS, social media | | HCPs |
| Health Alert Network[14, 41-43] | CDC | | | | USA (country) | | email, fax, web site | | HCPs, local and state HDs |
| Health Infoway[44] | Public Health Agency of Canada, Canada Health Infoway | | | | Canada  (country) | | email, phone, SMS, pager | | HCPs, hospitals, pharmacies |
| Hospital Emergency Response Data System (HERDS)[30] | NY State HD | | | | NY  (state) | | email, fax, phone | | Hospital HCPs, county health dept personnel |
| Integrated Health Alerting and Notification System (IHANS)[45] | NY State Health Commerce System | | | | NY  (state) | | email, fax, phone, web site | | Hospital HCPs, local health dept |
| Local HAN[46] | NY DOH, Western NY HDs in Buffalo | | | | Western NY State  (regional) | | email, fax | | MDs, hospitals, Emergency Depts |
| Local HAN[47] | Rochester Area Community Healthcare Information System (RACHIS) | | | | Monroe County, NY  (regional) | | fax, pager, phone, radio, "digital wireless devices" | | HCPs |
| local HAN[48] | Office of Public Health Emergency Preparedness (OPHEP) within the Maine Center for Disease Control and Prevention (Maine CDC) | | | | Maine  (state) | | email, fax | | HCPs, Emergency Depts |
| Massachusetts Homeland and Health Alert Network (HHAN)[14, 42, 49] | Massachusetts Department of Public Health | | | | MA  (state) | | email, SMS, fax, pager, phone, web site, other devices | | HCPs, Infection Control Practitioners |
| MHD Biosurveillance Dashboard[50] | Milwaukee Health Dept | | | | Milwaukee  (city) | | email, pager, web site | | Emergency Depts |
| Rapid Syndrome Validation Project (RSVP)[51] | Sandia/Los Alamos National Laboratories, University of NM, NM Dept of Health Office of Epidemiology | | | | New Mexico  (state) | | alert in EMR | | Emergency Depts |
| Rapid Syndrome Validation Project for Animals (RSVP-A)[52] | Sandia National Laboratories, KS University, NM State University, NM State Dept of Agriculture | | | | Kansas, New Mexico  (state) | | phone, mobile Palm | | veterinarians |
